# Shading Shapes Phyllosphere and Rhizosphere Bacterial Communities in Seedlings of the Karst Endangered Plant *Malania oleifera*

**DOI:** 10.3390/microorganisms14071421

**Published:** 2026-06-29

**Authors:** Yishan Yang, Rong Zou, Yunsheng Jiang, Yajin Luo, Zhenhai Deng, Shengfeng Chai, Jianmin Tang, Xiao Wei, Wenbin Guan

**Affiliations:** 1 School of Ecology and Nature Conservation, Beijing Forestry University, Beijing 100083, China; yangyishan0113@163.com; 2 Guangxi Key Laboratory of Plant Functional Substances and Resources Sustainable Utilization, Guangxi Institute of Botany, Guilin 541006, China; zr@gxib.cn (R.Z.); jys@gxib.cn (Y.J.); csf@gxib.cn (S.C.); 18877384841@163.com (J.T.); 3 Guangxi Yachang Orchid Conservation Area Management Center, Baise 533200, China; luoyajin320@163.com (Y.L.); 68368598@163.com (Z.D.)

**Keywords:** 16S rRNA amplicon sequencing, dominant microbial taxa, relative abundance, moderate shading, microbial agents

## Abstract

*Malania oleifera* is an endangered woody oil tree species endemic to China, where light conditions play a key role in seedling establishment. However, responses of seedling-associated bacterial communities to shading remain poorly characterized. This study investigated phyllosphere and rhizosphere bacterial communities under different shading regimes using 16S rRNA amplicon sequencing in two-year-old seedlings. Seedlings were subjected to 75%, 50%, and 25% shading, full-light conditions, and a field-grown reference group. The phyllosphere bacterial community showed lower alpha diversity and stronger compositional variation across shading treatments than the rhizosphere community, indicating higher sensitivity of leaf-associated bacteria to changes in the light environment. The rhizosphere maintained a larger shared bacterial pool and more stable community composition, with treatment-related differences mainly reflected in shifts in relative abundance. Functional prediction using PICRUSt2 indicated that phyllosphere bacterial functions were more responsive to shading than those in the rhizosphere, particularly pathways associated with genetic information processing and metabolism. PICRUSt2-based KEGG predictions suggested that both the phyllosphere and rhizosphere communities in the less-shaded and unshaded treatments were enriched for pathways related to aromatic compound degradation. Co-occurrence network analysis further revealed that phyllosphere association patterns were more sensitive to shading variation, whereas rhizosphere network complexity remained relatively stable. Shading exerted compartment-specific effects on the bacterial communities of *M. oleifera* seedlings, with the phyllosphere microbiota showing higher sensitivity to light variation than the rhizosphere communities.

## 1. Introduction

*Malania oleifera* (family Tapisciaceae; genus *Malania*) is an evergreen tall tree endemic to China and the only species within its genus. Its distribution is highly restricted, occurring only on limestone and adjacent earthy hillsides in southwestern China, primarily in the provinces of Guizhou, Guangxi, and Yunnan. Due to its narrow distribution range, small population size, and poor natural regeneration, *M. oleifera* has been listed as a National Class II protected plant in China [1,2] and categorized as Vulnerable in China’s Red List of Biodiversity—Higher Plants [3]. In addition to its conservation importance, *M. oleifera* has considerable economic value because its kernels are rich in nervonic acid, a long-chain monounsaturated fatty acid with nutritional and industrial applications [4,5]. Field observations indicate that natural regeneration of *M. oleifera* is generally weak, with few seedlings or saplings found beneath adult trees, and populations are often dominated by mature or old individuals. This age structure suggests that limited seedling recruitment is a major constraint on population renewal. Although previous studies have investigated its resource distribution [6], ecological and biological characteristics [7,8], genomic variation and genetic diversity [9,10], artificial propagation strategies [11,12], and nervonic acid accumulation [13], relatively little attention has been given to environmental factors regulating seedling establishment. In particular, the role of plant-associated microbial communities in seedling establishment remains largely unknown.

Light is a key environmental factor regulating plant growth, development, and photosynthetic metabolism. For endangered tree species with limited natural regeneration, appropriate light conditions during early development are particularly important for successful seedling establishment. Previous studies have shown that *M. oleifera* seedlings are sensitive to shading intensity. Xiong et al. [14] reported that seedling biomass initially increased and then decreased with increasing shading, with maximum biomass observed under moderate shading, suggesting that partial shading may alleviate light stress and enhance seedling acclimation. However, most existing studies have focused on morphological and physiological responses, while the potential role of plant-associated microbiota in mediating responses to light environments remains poorly understood. Light can influence plant-associated microbial communities through both direct and indirect mechanisms. In the phyllosphere, variation in light intensity can alter leaf surface temperature, humidity, ultraviolet exposure, and the release of leaf-derived metabolites, reshaping bacterial colonization and community structure. In the rhizosphere, although light does not directly act on soil microorganisms, it can regulate microbial communities indirectly by changing photosynthetic carbon allocation, root growth, and root exudation patterns [15]. Recent studies have demonstrated that light intensity alters phyllosphere microbial composition and functional profiles in lettuce [16], while reduced photosynthetically active radiation perceived by shoots can reshape root-associated bacterial communities in *Arabidopsis thaliana* [17,18]. These results indicate that aboveground light conditions may exert coordinated effects on both leaf- and root-associated microbiota. However, current microbiome research on *M. oleifera* has primarily focused on comparisons across natural habitats [19,20], differences between healthy and diseased individuals [21], and endophytic fungal diversity [22]. The responses of phyllosphere and rhizosphere bacterial communities to shading gradients in seedlings remain unclear, and it is not yet known whether above- and belowground bacterial communities respond similarly or differently under varying light conditions. This knowledge gap limits our understanding of how shading management can shape the microbial environment during *M. oleifera* seedling establishment.

Therefore, a nursery-based shading experiment was established using two-year-old seed-grown *M. oleifera* seedlings, with age-matched field-grown plants included as a natural reference. Unlike previous studies focusing on single plant compartments or natural habitat comparisons, this study simultaneously characterized phyllosphere and rhizosphere bacterial communities from the same seedlings along a defined shading gradient (75%, 50%, 25%, and 0% shading). This paired design enabled direct comparison of above- and belowground bacterial communities under controlled environmental conditions and allowed assessment of whether these two microbial compartments respond similarly or in a compartment-specific manner to shading. The objectives of this study were to: (1) determine how bacterial diversity and community composition respond to changes in light availability; (2) identify key taxa and predicted functional profiles associated with different shading treatments; and (3) compare whether phyllosphere and rhizosphere bacterial communities exhibit similar or distinct responses to shading. This work provides a microecological perspective on how light environments shape plant-associated bacterial communities in *M. oleifera* seedlings and offers insights for optimizing nursery cultivation and seedling management strategies.

## 2. Materials and Methods

### 2.1. Study Site

The experiment was carried out in a nursery at the Guangxi Institute of Botany, Guilin, Guangxi Zhuang Autonomous Region, China (110°17′ E, 25°01′ N). The region has a mid-subtropical monsoon climate, with a mean annual temperature of 19.2 °C. The mean monthly temperatures in January, the coldest month, and July, the warmest month, are 8.4 °C and 28.4 °C, respectively [23].

### 2.2. Plant Materials and Growth Substrate

The growth substrate used in the shading experiment was collected in May 2024 from three wild populations within the natural distribution range of *M. oleifera* in Leye County, Baise City, Guangxi Zhuang Autonomous Region, China. In each population, five sampling points were randomly selected near naturally occurring *M. oleifera* individuals. After removal of the surface litter layer, soil was collected from the 20–30 cm depth, thoroughly homogenized, and transported to the experimental site for use as a common potting substrate. Two-year-old, seed-derived *M. oleifera* seedlings were used as the experimental materials. After transplantation into the native soil substrate, the seedlings were allowed to acclimate for two months before the shading treatments were initiated.

### 2.3. Experimental Design and Shading Treatments

After acclimation, uniform and healthy seedlings without visible pests or disease symptoms were assigned to four light treatments: L1, 75% shading; L2, 50% shading; L3, 25% shading; and L4, 0% shading, representing full sunlight. Each treatment consisted of 15 seedlings, which were randomly arranged within the corresponding treatment area. The shading treatments were established using commercial black shade nets. Before the treatments began, photosynthetically active radiation (PAR) under full sunlight was measured at seedling canopy height and used as the reference value. The target PAR levels for L1, L2, and L3 were set at approximately 25%, 50%, and 75% of the full-light PAR, respectively. Shade nets were then installed and adjusted until the measured PAR at canopy height reached the target level for each treatment, while L4 was left unshaded. The top and sides of each treatment area were enclosed to minimize lateral light interference. Irrigation, fertilization, and routine nursery management were kept consistent across all treatments throughout the experiment.

The shading experiment was initiated in August 2024, and phyllosphere and rhizosphere samples were collected after 45 days of treatment. This sampling time was chosen to assess short-term responses of bacterial communities to contrasting light environments while minimizing potential confounding effects related to seasonal changes. At the end of the experiment, three seedlings were randomly selected from each treatment for destructive microbial sampling and treated as independent biological replicates. The remaining seedlings were maintained under the same treatment conditions but were not included in the sequencing analysis. In addition to the nursery shading treatments, three field-grown *M. oleifera* seedlings of the same age were selected from a natural population as a field-grown reference group. These field-grown plants were sampled using the same procedures as the nursery-grown seedlings. This group was included only as a descriptive natural reference and was not considered a controlled shading treatment.

To characterize the actual light environment during the experiment, PAR was measured at seedling canopy height on clear days using an LI-190R Quantum Sensor (LI-COR Biosciences, Lincoln, NE, USA). The mean PAR values were approximately 275, 550, 825, and 1100 μmol m^−2^ s^−1^ under L1, L2, L3, and L4, respectively. Air temperature within each treatment area was maintained at 28 ± 2 °C, and soil moisture was regularly monitored and maintained at approximately 60–70% of field water capacity through controlled irrigation. These measures were used to reduce the potential influence of temperature and soil water availability on variation in bacterial communities.

### 2.4. Collection of Phyllosphere and Rhizosphere Samples

Each shading treatment included 15 seedlings. At the end of the experiment, seedlings that were healthy and relatively uniform in growth were first screened, after which three seedlings were randomly selected from each treatment for destructive microbial sampling and treated as independent biological replicates [24]. For each biological replicate, five fully expanded, healthy mature leaves were collected from the upper and middle canopy of a single seedling and pooled as one phyllosphere sample. Leaves with visible lesions, insect damage, or dust contamination were avoided. All samples were collected on the same day between 9:00 and 10:00 to minimize potential effects of diurnal variation. Sterile gloves, scissors, and forceps were used throughout the sampling procedure. Immediately after collection, the leaf samples were placed in sterile 50 mL centrifuge tubes, transported to the laboratory on ice, and processed within 2 h. In the laboratory, pre-chilled sterile PBS buffer (1× PBS, pH approximately 7.4) was added to each tube, and the samples were shaken at 200 rpm for 15–20 min to detach epiphytic microorganisms from the leaf surface [25]. The wash suspension was first filtered to remove plant debris and then centrifuged to collect microbial cells. The supernatant was discarded, and the resulting microbial pellet was stored at −80 °C until DNA extraction and sequencing.

Rhizosphere soil was collected from the same seedlings used for phyllosphere sampling. Seedlings were carefully removed from the pots, and loosely attached soil was gently shaken off. Soil tightly adhering to the root surface was then collected using sterile brushes and forceps and pooled as one rhizosphere sample for each seedling. Approximately 1.0 g of rhizosphere soil was collected from each seedling, transferred into a sterile 5.0 mL centrifuge tube [26], immediately frozen in liquid nitrogen, transported to the laboratory, and stored at −80 °C until DNA extraction.

### 2.5. DNA Extraction, PCR Amplification, and Sequencing

Microbial genomic DNA was extracted from phyllosphere and rhizosphere samples using the E.Z.N.A.^®^ Soil DNA Kit (Omega Bio-tek, Norcross, GA, USA) according to the manufacturer’s instructions. The extracted DNA was used as the template for amplification of the bacterial 16S rRNA gene [27]. To reduce the co-amplification of plant-derived organellar sequences, a two-step PCR strategy was adopted. In the first round, the bacterial 16S rRNA gene fragment was amplified using primers 799F (5′-AACMGGATTAGATACCCKG-3′) and 1392R (5′-ACGGGCGGTGTGTRC-3′). In the second round, the purified first-round PCR products were used as templates and re-amplified using primers 799F (5′-AACMGGATTAGATACCCKG-3′) and 1193R (5′-ACGTCATCCCCACCTTCC-3′), together with sample-specific barcodes.

Each PCR was performed in a 20 μL reaction mixture containing 4 μL of 5× FastPfu Buffer, 2 μL of 2.5 mM dNTPs, 0.8 μL of forward primer (5 μM), 0.8 μL of reverse primer (5 μM), 0.4 μL of FastPfu DNA Polymerase, 0.2 μL of BSA, approximately 10 ng of template DNA, and nuclease-free water to a final volume of 20 μL. FastPfu DNA Polymerase, 5× FastPfu Buffer, dNTPs, BSA, and nuclease-free water were obtained from TransGen Biotech Co., Ltd. (Beijing, China), and primers were synthesized by Sangon Biotech Co., Ltd. (Shanghai, China). The PCR cycling conditions were as follows: initial denaturation at 95 °C for 3 min; followed by denaturation at 95 °C for 30 s, annealing at 55 °C for 30 s, and extension at 72 °C for 45 s; and a final extension at 72 °C for 10 min. PCR products were examined by 2% agarose gel electrophoresis, purified using an AxyPrep DNA Gel Extraction Kit (Axygen Biosciences, Union City, CA, USA), and quantified using a QuantiFluor-ST fluorometer (Promega Corporation, Madison, WI, USA) before library construction. Purified amplicons were pooled in equimolar amounts and subjected to paired-end sequencing on an Illumina MiSeq PE300 platform (Illumina, Inc., San Diego, CA, USA) by Shanghai Majorbio Bio-Pharm Technology Co., Ltd. (Shanghai, China).

Several steps were taken to reduce PCR-related amplification bias. First, a consistent amount of high-quality template DNA was used for each sample, and all reactions were performed using the same reaction system and cycling conditions. Second, FastPfu high-fidelity DNA polymerase was used to improve amplification accuracy. Third, the number of PCR cycles was kept to the minimum required to obtain sufficient amplicon yield, thereby reducing the risk of over-amplification. Fourth, PCR products were purified and quantified before pooling, and amplicons from different samples were combined in equimolar amounts for sequencing. Negative controls were included during PCR amplification to monitor potential contamination.

### 2.6. Sequence Processing and Taxonomic Annotation

Raw reads were processed using fastp v0.19.6 (OpenGene, Shenzhen, China) to generate sequence summaries, perform quality filtering, and trim low-quality bases. Paired-end reads were subsequently merged using FLASH v1.2.11 (Center for Computational Biology, Johns Hopkins University, Baltimore, MD, USA). Operational taxonomic unit (OTU) clustering was performed with UPARSE v11 (drive5, Tiburon, CA, USA) at 97% sequence similarity. The 97% OTU-based workflow was retained to ensure consistency with the standardized UPARSE pipeline used for the initial sequence processing and downstream analyses. According to shading level and sample type, phyllosphere samples were assigned to the L1_L, L2_L, L3_L, L4_L, and W_L groups, whereas rhizosphere samples were assigned to the L1_RS, L2_RS, L3_RS, L4_RS, and W_RS groups.

### 2.7. Diversity, Differential Taxa, and Functional Prediction Analyses

The OTU table generated after sequence processing was used for downstream microbial community analyses on the Majorbio Cloud Platform (Shanghai Majorbio Bio-Pharm Technology Co., Ltd., Shanghai, China) and in R version 4.4.3 (R Foundation for Statistical Computing, Vienna, Austria). To minimize the influence of unequal sequencing depth among samples, the OTU count table was rarefied by random subsampling to 38,132 sequences per sample, which corresponded to the lowest sequencing depth among the retained samples after quality filtering. Alpha-diversity indices, including the Shannon and Chao indices, as well as Venn diagrams, Bray–Curtis dissimilarity, PCoA, PERMANOVA, and PERMDISP analyses, were performed using the rarefied OTU table.

For taxonomic composition, PCA, LEfSe analysis, and visualization of predicted functional profiles, OTU counts or predicted functional abundances were transformed into relative abundances by total-sum scaling, whereby each feature count was divided by the total count of the corresponding sample. Cumulative-sum scaling normalization was not applied. PCA was used as an unconstrained exploratory ordination method to visualize overall variation in bacterial community composition or predicted functional profiles among treatments. Because PCA was used only for visualization, treatment-related differences in bacterial community composition were further tested using PERMANOVA based on Bray–Curtis dissimilarity with 999 permutations. PCoA analyses were conducted separately for phyllosphere and rhizosphere bacterial communities, with field-grown samples included as a natural reference group. Homogeneity of multivariate dispersion among groups was assessed using PERMDISP.

LEfSe analysis was conducted separately for phyllosphere and rhizosphere samples to identify bacterial taxa associated with different shading treatments. Taxa with *p* < 0.05 and an LDA score > 4.0 were retained and displayed using cladograms and LDA score plots. This relatively stringent LDA threshold was applied to emphasize taxa with strong discriminatory effects among treatments.

Potential bacterial functions were predicted using PICRUSt2 based on the 16S rRNA OTU representative sequences and the corresponding OTU abundance table, and were summarized against the COG and KEGG databases [28]. The predicted functional profiles were converted to relative abundances for comparison and visualization among treatments. KEGG level-3 pathways showing evident variation among treatments were visualized using heatmaps and bubble plots in R version 4.4.3. To assess the reliability of PICRUSt2-based functional predictions, the weighted nearest sequenced taxon index (weighted NSTI) was calculated for each sample. Lower weighted NSTI values indicate closer phylogenetic relationships between the observed taxa and available reference genomes and therefore generally reflect higher confidence in the predicted functional profiles. Weighted NSTI values were summarized separately for phyllosphere and rhizosphere samples and are provided in Table S3.

For co-occurrence analysis, networks were constructed separately for phyllosphere and rhizosphere communities under each treatment based on the relative-abundance OTU table to visualize association patterns among bacterial OTUs. Pairwise associations were estimated using Spearman’s rank correlation, and only correlations with |r| > 0.6 and *p* < 0.05 were retained. Network parameters and visualizations were generated in R.

### 2.8. Statistical Analysis

Statistical analyses were conducted in R version 4.4.3 (R Foundation for Statistical Computing, Vienna, Austria). The main R packages used in this study included vegan for Bray–Curtis dissimilarity, PCoA, PERMANOVA, and PERMDISP analyses; ggplot2 for data visualization; dplyr and tidyr for data processing; car for Levene’s test; dunn.test for Dunn’s post hoc test; pheatmap for heatmap visualization; and igraph and ggraph for co-occurrence network analysis and visualization.

Before comparisons of alpha-diversity indices and other univariate variables among treatments, data were tested for normality and homogeneity of variance using the Shapiro–Wilk test and Levene’s test, respectively. When both assumptions were met, differences among treatments were assessed using one-way analysis of variance followed by Tukey’s honestly significant difference test for multiple comparisons. When these assumptions were not satisfied, the Kruskal–Wallis test followed by Dunn’s post hoc test was used. For post hoc pairwise comparisons, *p*-values were adjusted using the Benjamini–Hochberg false discovery rate method where appropriate. Unless otherwise stated, differences were considered statistically significant at *p* < 0.05.

## 3. Results

### 3.1. Alpha-Diversity Patterns of Phyllosphere and Rhizosphere Bacterial Communities Under Shading Treatments

**Figure 1:**
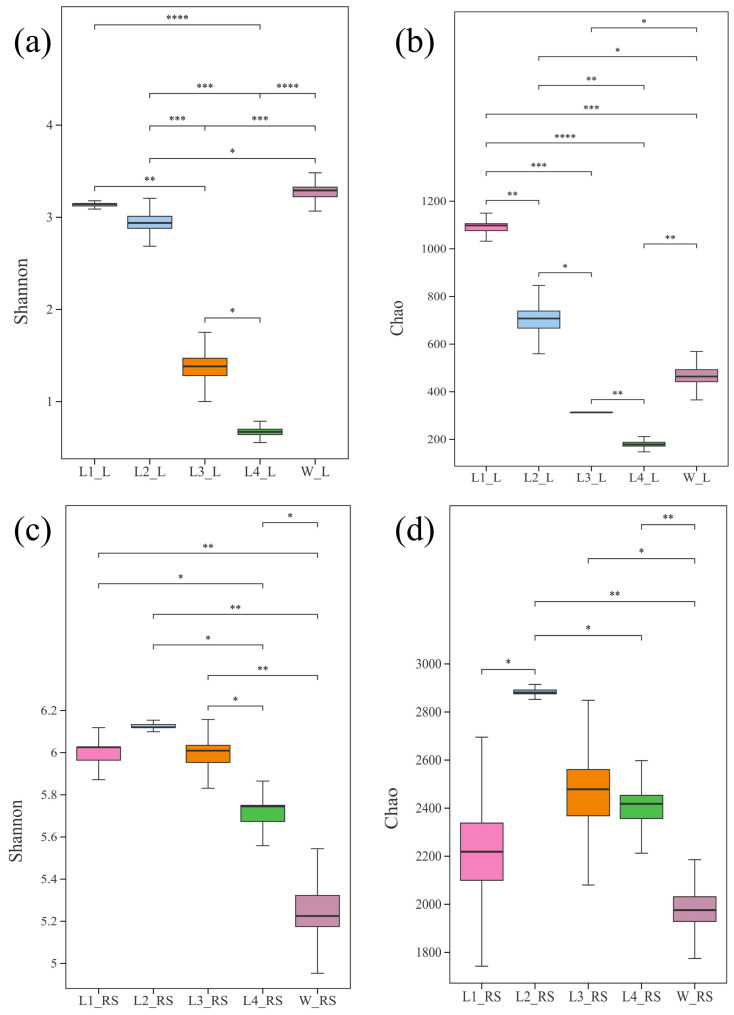
Alpha-diversity of bacterial communities in the phyllosphere and rhizosphere of *Malania oleifera* seedlings under different shading treatments. Shannon and Chao indices were used to evaluate bacterial diversity and richness, respectively, in the phyllosphere (**a**,**b**) and rhizosphere (**c**,**d**). L1, L2, L3, and L4 represent 75%, 50%, 25%, and 0% shading, respectively. W_L and W_RS represent field-grown phyllosphere and rhizosphere samples used as natural references. The same abbreviations apply below. *, **, ***, and **** indicate significant differences at *p* < 0.05, *p* < 0.01, *p* < 0.001, and *p* < 0.0001, respectively.

Shannon and Chao indices were used to evaluate bacterial diversity and richness in the phyllosphere and rhizosphere under different shading treatments (Figure 1). Exact *p*-values for significant pairwise differences among treatment groups are provided in Tables S1 and S2. In the phyllosphere, both indices generally declined as shading intensity decreased. The Shannon index was relatively high in L1_L and W_L, whereas L4_L showed the lowest diversity (Figure 1a). A similar pattern was observed for the Chao index, with greater richness under stronger shading and a pronounced reduction under higher light exposure, particularly in L3_L and L4_L (Figure 1b).

Compared with the phyllosphere, the rhizosphere bacterial community showed higher alpha diversity and less variation among shading treatments (Figure 1c,d). The Shannon index remained relatively stable in L1_RS, L2_RS, and L3_RS, but declined in L4_RS and W_RS. For the Chao index, L2_RS exhibited the highest richness, followed by L3_RS and L1_RS, whereas W_RS had the lowest value. These results suggest that phyllosphere bacterial alpha diversity was more responsive to changes in light conditions, whereas rhizosphere bacterial communities maintained relatively higher and more stable diversity across the shading gradient. Notably, moderate shading was associated with relatively high bacterial richness, particularly in the rhizosphere.

### 3.2. OTU Distribution and Beta-Diversity Patterns of Phyllosphere and Rhizosphere Bacterial Communities

**Figure 2:**
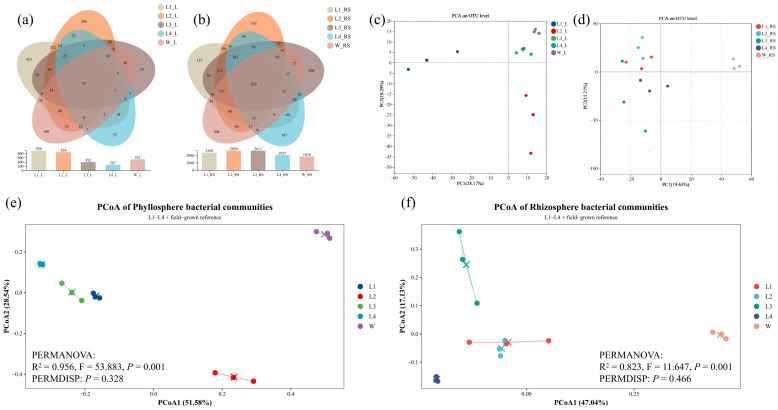
Venn diagrams (**a**,**b**), PCA (**c**,**d**), and PCoA analyses (**e**,**f**) showing differences in the composition and beta diversity of phyllosphere and rhizosphere bacterial communities of *Malania oleifera* seedlings under different shading treatments.

Venn analysis revealed distinct OTU distribution patterns between phyllosphere and rhizosphere bacterial communities under different shading treatments (Figure 2). In the phyllosphere, only 73 OTUs were shared among the five groups, indicating a relatively small common bacterial pool on the leaf surface (Figure 2a). The more heavily shaded treatments, particularly L1_L and L2_L, contained higher total numbers of OTUs and more unique OTUs than the less shaded treatments, L3_L and L4_L. By contrast, OTU numbers decreased under stronger light exposure, and W_L also differed from the artificial shading treatments in OTU composition. PCA further showed clearer separation among phyllosphere samples from different treatments, indicating marked variation in phyllosphere bacterial composition across light conditions (Figure 2c). This pattern was supported by PERMANOVA, which detected a significant treatment effect on phyllosphere bacterial community composition when all five groups were included (R^2^ = 0.956, F = 53.883, *p* = 0.001) (Figure 2e). PERMDISP was not significant (*p* = 0.291), suggesting that the observed separation was mainly attributable to differences in community composition rather than differences in within-group dispersion (Figure 2e).

In the rhizosphere, 833 OTUs were shared among the five groups, far exceeding the number shared in the phyllosphere (Figure 2b). Total OTU numbers ranged from 1830 to 2630 across treatments, with the highest values observed in L2_RS and L3_RS and the lowest value in W_RS. Unlike the phyllosphere, PCA showed only weak separation among rhizosphere samples from the artificial shading treatments, whereas W_RS was more clearly separated from the nursery treatments (Figure 2d). PERMANOVA also detected a significant treatment effect on rhizosphere bacterial community composition when all five groups were included (R^2^ = 0.823, F = 11.647, *p* = 0.001), consistent with the separation of W_RS from the nursery treatments. PERMDISP was not significant (*p* = 0.466), indicating that the significant PERMANOVA result was not driven by unequal dispersion among groups. These results suggest that the rhizosphere bacterial community retained a larger shared OTU pool and showed less pronounced variation across shading treatments than the phyllosphere community. Overall, the Venn, PCA, PERMANOVA, and PERMDISP results consistently indicate that phyllosphere bacterial communities were more responsive to changes in light conditions, whereas rhizosphere communities maintained a relatively more stable composition across the shading gradient.

### 3.3. Bacterial Community Composition in the Phyllosphere and Rhizosphere of M. oleifera Seedlings Under Different Shading Environments

**Figure 3:**
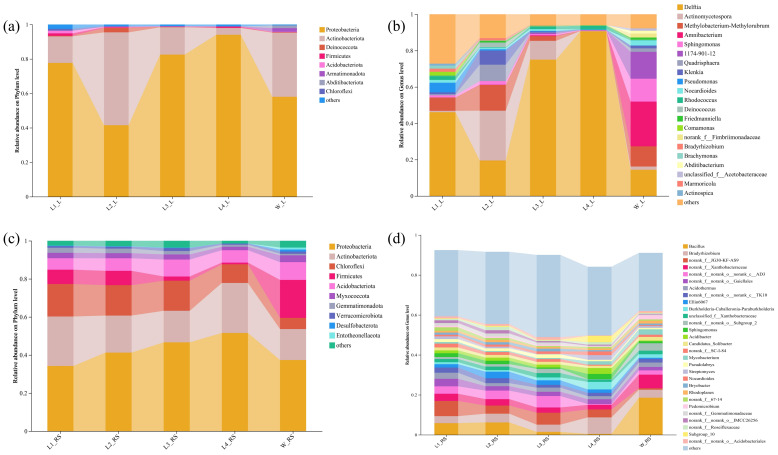
Composition of bacterial communities in the phyllosphere (**a**,**b**) and rhizosphere (**c**,**d**) of *Malania oleifera* seedlings under different shading treatments.

The phylum- and genus-level compositions of phyllosphere and rhizosphere bacterial communities under different shading treatments are shown in Figure 3. In the phyllosphere, Proteobacteria was the dominant phylum, followed by Actinobacteriota. Proteobacteria predominated in L1_L, L3_L, and L4_L, whereas the relative abundance of Actinobacteriota was comparatively low in these treatments. By contrast, Actinobacteriota showed a higher relative abundance in L2_L. Firmicutes, Deinococcota, and Acidobacteriota occurred at low relative abundances but showed slight variation among treatments.

Genus-level analysis further resolved changes in dominant taxa. In most artificial shading treatments, including L1_L, L3_L, and L4_L, *Delftia* showed the highest relative abundance and represented a typical dominant genus. In L2_L, however, the dominance of *Delftia* was reduced, whereas the relative abundances of actinobacterial genera such as *Actinomycetospora*, *Quadrisphaera*, and *Klenkia* increased, resulting in a more dispersed community composition. In the field-grown reference group W_L, the relative abundances of genera such as *Amnibacterium*, *Sphingomonas*, and 1174-901-12 increased markedly. These results indicate that the phyllosphere bacterial community responded to shading mainly through shifts in dominant genera, rather than through a uniform increase or decrease across all major taxa.

At the phylum level, the rhizosphere bacterial community showed a more balanced distribution pattern (Figure 3c). Proteobacteria and Actinobacteriota remained the two dominant phyla, whereas Chloroflexi, Firmicutes, Acidobacteriota, Gemmatimonadota, and other phyla had markedly higher relative abundances than in the phyllosphere and varied among shading treatments. Under L2_RS and L3_RS, the relative abundances of Acidobacteriota and Gemmatimonadota increased, whereas Proteobacteria was more abundant in L4_RS.

At the genus level, the rhizosphere bacterial community consisted of numerous genera with low to moderate relative abundances (Figure 3d). Typical plant-associated genera, such as *Bacillus*, *Bradyrhizobium*, and *Streptomyces*, were consistently detected across all treatments, with only minor differences in relative abundance among treatments. In contrast, many “norank” taxa and genera that could not be precisely assigned showed greater variation among treatments. The relative abundances of some “norank” genera and *Candidatus solibacter* varied across shading treatments, whereas Proteobacteria-associated genera were more abundant in W_RS. Unlike in the phyllosphere, shifts among dominant rhizosphere genera were relatively limited.

Overall, the phyllosphere bacterial community showed more apparent shifts in dominant taxa across shading treatments, especially between Proteobacteria- and Actinobacteriota-related groups. By contrast, the rhizosphere bacterial community showed a more even composition, with treatment-related changes mainly reflected in the relative abundances of multiple low- to moderate-abundance taxa.

### 3.4. Differential Taxa in Phyllosphere and Rhizosphere Bacterial Communities Under Different Shading Treatments

**Figure 4:**
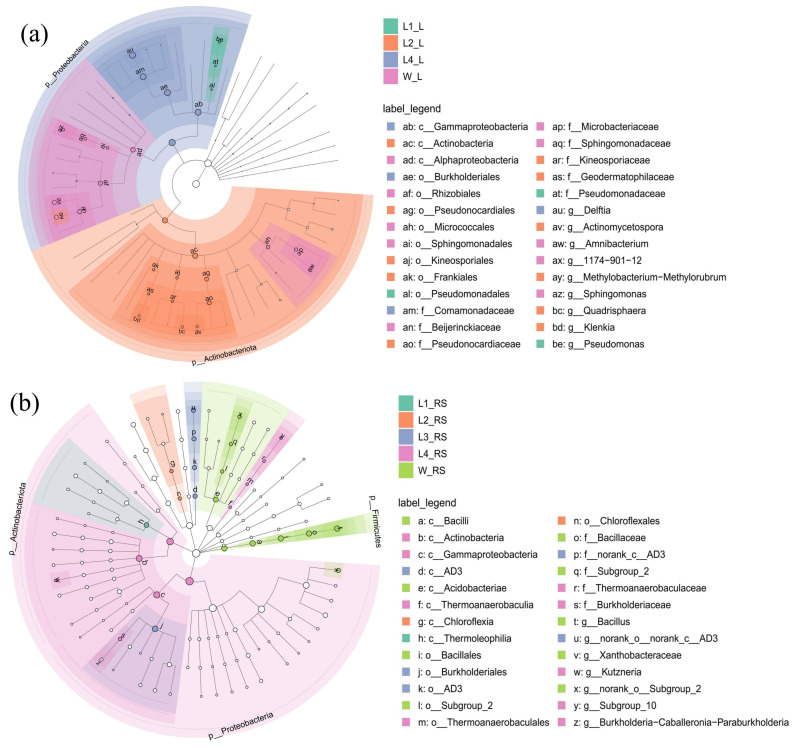
LEfSe analysis of differentially abundant bacterial taxa among phyllosphere (**a**) and rhizosphere (**b**) communities of *Malania oleifera* seedlings under different shading treatments. Only taxa meeting the criteria of *p* < 0.05 and LDA score > 4.0 are shown.

LEfSe analysis was used to identify bacterial taxa that differed among shading treatments, with an LDA score > 4.0 used as the screening threshold (Figure 4). In the phyllosphere, most differential taxa belonged to Proteobacteria and Actinobacteriota (Figure 4a). Under full light (L4_L), several Proteobacteria-related taxa were enriched, including Gammaproteobacteria, Burkholderiales, Comamonadaceae, and *Delftia*. In L1_L, Pseudomonadales, Pseudomonadaceae, and Pseudomonas, which were more abundant. By contrast, L3_L did not contain any treatment-associated bacterial taxa that met the LEfSe screening criteria of *p* < 0.05 and LDA score > 4.0.

In W_L, Alphaproteobacteria and related taxa, including Sphingomonadales and the Rhizobiales group 1174-901-12, were enriched. The order Micrococcales within Actinobacteriota was also enriched in W_L. In L2_L, *Methylobacterium–Methylorubrum*, *Quadrisphaera*, *Klenkia*, and *Actinomycetospora* showed higher relative abundances than in the other phyllosphere groups. These results indicate that different shading treatments were associated with distinct phyllosphere indicator taxa across multiple taxonomic levels.

The rhizosphere showed a LEfSe pattern distinct from that of the phyllosphere (Figure 4b). In L4_RS, *Kutzneria*, the *Burkholderia–Caballeronia–Paraburkholderia* complex, and Subgroup_10 within Thermoanaerobaculia were enriched. In L1_RS, Thermoleophilia was the main enriched taxon. The two moderate-shading treatments also differed in their enriched taxa: L2_RS was enriched in Chloroflexia and Chloroflexales, whereas L3_RS was characterized by AD3-related taxa. In W_RS, Firmicutes, Acidobacteriae, Xanthobacteraceae, and related taxa were enriched.

Overall, LEfSe analysis showed that the differential taxa in the phyllosphere were mainly concentrated within Proteobacteria and Actinobacteriota, whereas the rhizosphere contained a broader range of differential taxa across Proteobacteria, Firmicutes, Acidobacteriota, Chloroflexi, and other groups. Compared with the rhizosphere, the phyllosphere showed more distinct treatment-associated shifts in dominant bacterial lineages.

### 3.5. COG Functional Prediction of Phyllosphere and Rhizosphere Bacteria Under Different Shading Treatments

To assess the reliability of PICRUSt2-based functional predictions, weighted NSTI values were calculated for all samples and are summarized in Table S3. The mean weighted NSTI values ranged from 0.051 to 0.130 in the phyllosphere and from 0.220 to 0.332 in the rhizosphere, indicating greater reference-genome representation, and thus higher prediction confidence, for phyllosphere communities.

**Figure 5:**
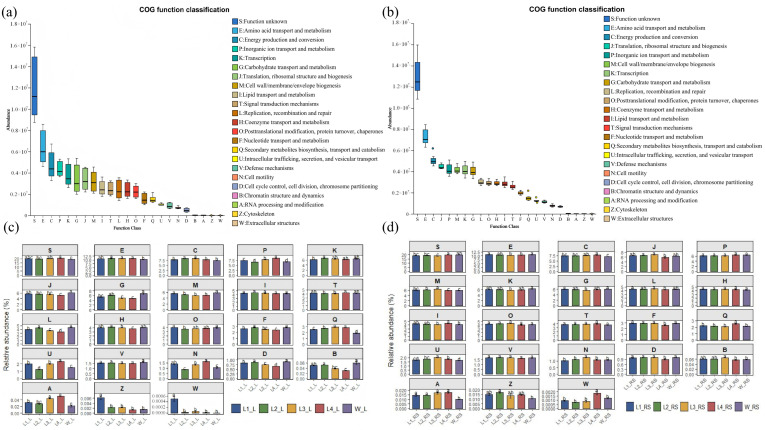
COG functional prediction of phyllosphere and rhizosphere bacterial communities of *Malania oleifera* seedlings under different shading treatments. (**a**) Overall COG functional abundance in phyllosphere samples; (**b**) overall COG functional abundance in rhizosphere samples; (**c**) relative abundance of COG functional categories in phyllosphere samples; (**d**) relative abundance of COG functional categories in rhizosphere samples. Different lowercase letters above the bars indicate significant differences among groups within the same COG functional category (*p* < 0.05).

COG functional profiles were then inferred from the 16S rRNA amplicon data using PICRUSt2 to characterize the potential functional capacity of phyllosphere and rhizosphere bacterial communities in *M. oleifera* seedlings under different light conditions (Figure 5). Overall, the phyllosphere and rhizosphere showed broadly similar predicted functional compositions. In both habitats, category S (function unknown) was the most abundant, followed by categories associated with amino acid metabolism, energy production, inorganic ion transport, and transcription.

To examine treatment-related functional variation, COG categories were compared among treatments (Figure 5c,d). In the phyllosphere, the main variable categories were associated with genetic information processing (J, K, L), signal transduction and cell structure (T, M, N), and energy and material metabolism (C, G, E, F, P, I). Category J showed only minor differences among treatments, although its relative abundance was higher in W_L than in the shading treatments and lowest in L4_L. Categories K and L were relatively more abundant in W_L and L2_L. Category T was most abundant in L4_L, whereas category M was highest in W_L and generally decreased as shading intensity declined among the shading treatments. Category N varied only slightly, with relatively higher values in W_L and L2_L. Among the metabolic categories, category C was more abundant in L2_L and L3_L. Category G was highest in W_L, followed by L2_L, and lowest in L4_L. Category E showed limited variation among the shading treatments, although all shaded treatments had higher values than W_L. Category F was relatively abundant in L2_L and W_L, whereas category P was enriched in L4_L. Category I showed little variation among treatments.

Compared with the phyllosphere, rhizosphere COG categories showed less variation among treatments. The predicted rhizosphere functional categories could also be grouped into genetic information processing (J, K, F, L), signal transduction and cell structure (T, M, O), and energy and material metabolism (C, G, E, I). Category J showed an increase followed by a decrease as shading intensity declined, with the highest value in L3_RS and the lowest value in L4_RS. Category K was more abundant in W_RS than in the shaded treatments and decreased gradually with decreasing shading intensity, whereas F and L showed no clear differences. Category T increased as shading intensity decreased and was highest in L4_RS. Categories M and O were more abundant in L3_RS than in the other treatments. Among the metabolic categories, category C increased as shading intensity decreased and was highest in L4_RS. Category G showed a decrease followed by an increase, with the lowest value in L3_RS and higher values in W_RS than in the shaded treatments. Category E decreased gradually with decreasing shading intensity among the shaded treatments, while W_RS remained higher. Category I also showed a decrease followed by an increase, with the highest value in L4_RS, the lowest value in L3_RS, and an intermediate value in W_RS.

Overall, the predicted COG profiles showed greater treatment-related variation in the phyllosphere than in the rhizosphere. In the phyllosphere, variation was mainly observed in categories related to genetic information processing and energy/material metabolism. In the rhizosphere, treatment-related differences were smaller and were mainly reflected in categories associated with energy metabolism, signal transduction, and cell-structure-related functions.

### 3.6. KEGG Functional Prediction of Phyllosphere and Rhizosphere Bacteria Under Different Shading Treatments

**Figure 6:**
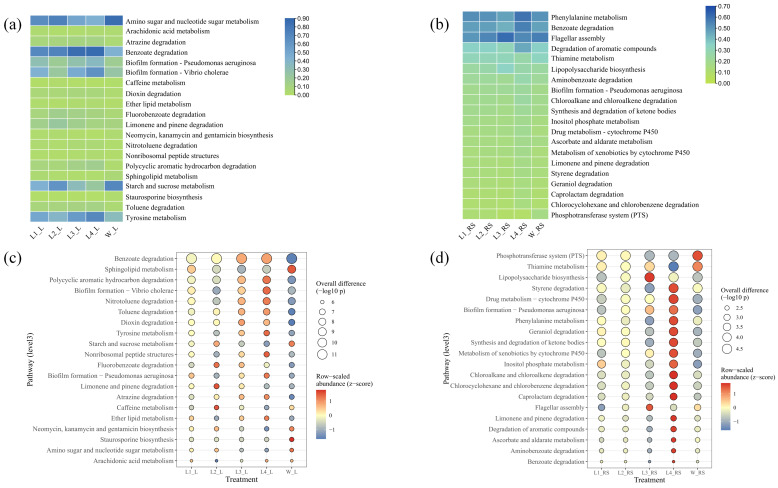
Predicted KEGG level-3 functional profiles of phyllosphere and rhizosphere bacterial communities in *Malania oleifera* seedlings under different shading treatments. Heatmaps of selected KEGG level-3 pathways in the phyllosphere (**a**) and rhizosphere (**b**), respectively; bubble plots of the corresponding pathways in the phyllosphere (**c**) and rhizosphere (**d**), respectively, after row-wise standardization. In the heatmaps, color intensity indicates the relative predicted abundance of each pathway. In the bubble plots, bubble color represents the relative enrichment level of each treatment compared with the mean value of that pathway, and bubble size represents −log10(P).

At KEGG level 3, the predicted functional pathways of phyllosphere and rhizosphere bacterial communities were compared among shading treatments (Figure 6). The top 20 pathways showing treatment-related variation were selected for visualization. For each pathway, the abundances of contributing taxa were summarized and presented as heatmaps (Figure 6a,b). To further compare pathway variation among treatments, pathway abundances were row-standardized and displayed as bubble plots (Figure 6c,d).

For the phyllosphere community (Figure 6a), several pathways showed relatively high predicted abundances, including Amino sugar and nucleotide sugar metabolism, Benzoate degradation, Starch and sucrose metabolism, and Tyrosine metabolism. Across the selected pathways, the artificial shading treatments generally showed higher predicted enrichment than W_L (Figure 6c). Among the shading treatments, pathways related to Benzoate degradation, Toluene degradation, Dioxin degradation, and Atrazine degradation increased with decreasing shading intensity. These patterns suggest a higher predicted representation of aromatic compound degradation-related pathways in the less shaded and unshaded treatments.

Compared with the phyllosphere, the rhizosphere functional profile showed a weaker overall response to shading treatments (Figure 6b,d). Phenylalanine metabolism, Benzoate degradation, Flagellar assembly, and Degradation of aromatic compounds were among the pathways with relatively high predicted abundances in the rhizosphere (Figure 6b). As shown in Figure 6d, several pathways, including Phenylalanine metabolism, Benzoate degradation, and Degradation of aromatic compounds, reached their highest predicted abundances in L4_RS. In W_RS, Phosphotransferase system (PTS) and Thiamine metabolism showed relatively higher enrichment than most other pathways. Overall, KEGG prediction indicated that the less shaded and unshaded treatments were associated with increased predicted abundances of pathways related to aromatic compound degradation, particularly in the rhizosphere.

### 3.7. Co-Occurrence Networks of Phyllosphere and Rhizosphere Bacterial Communities

**Figure 7:**
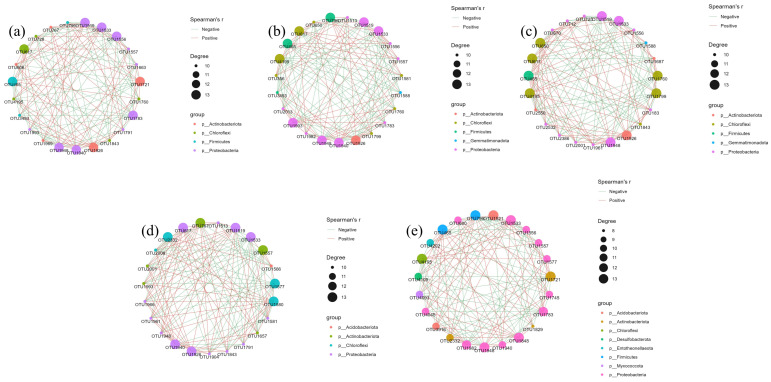
Co-occurrence networks of phyllosphere bacterial communities in *Malania oleifera* seedlings under different shading treatments. (**a**–**e**) represent L1_L, L2_L, L3_L, L4_L, and W_L, respectively. Nodes represent bacterial OTUs, and node colors indicate bacterial phyla. Node size is proportional to degree. Edges represent significant Spearman correlations between OTUs, with red and green lines indicating positive and negative correlations, respectively.

Co-occurrence networks of the phyllosphere bacterial community under different shading treatments are shown in Figure 7. The number and direction of significant edges varied among treatments. In L1_L, the numbers of positive and negative edges were equal (54/54), and W_L showed a similar balance between positive and negative associations (55/54). L2_L had the largest number of significant edges, with slightly more positive than negative edges (64/56). In L3_L, the total number of edges decreased, but positive edges still accounted for a larger proportion (55/34). By contrast, L4_L contained more negative than positive edges (37/51), indicating a shift in the association pattern of phyllosphere bacterial taxa under full-light conditions.

Proteobacteria-related taxa occurred as highly connected nodes in all phyllosphere networks. W_L contained a broader range of hub taxa at the phylum level than the artificial shading treatments. Among the shading treatments, both L1_L and L4_L had highly connected nodes spanning four phyla. In L4_L, Firmicutes was no longer detected as a hub taxa at the phylum level, whereas an Acidobacteriota taxon was identified as a highly connected node. In addition, L2_L and L3_L each contained a treatment-associated hub taxon belonging to Gemmatimonadota.

**Figure 8:**
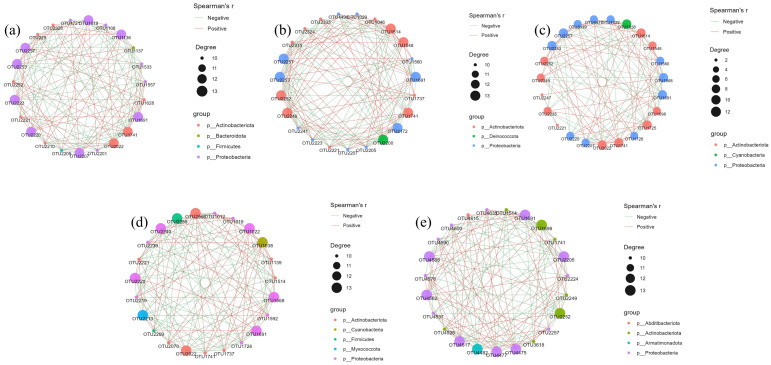
Co-occurrence networks of rhizosphere bacterial communities in *Malania oleifera* seedlings under different shading treatments. Panels (**a**–**e**) represent L1_RS, L2_RS, L3_RS, L4_RS, and W_RS, respectively. Nodes represent bacterial OTUs, and node colors indicate bacterial phyla. Node size is proportional to degree. Edges represent significant Spearman correlations between OTUs, with red and green lines indicating positive and negative correlations, respectively.

Co-occurrence networks of the rhizosphere bacterial community are shown in Figure 8. All rhizosphere networks contained 23 nodes, but the number of significant edges varied among treatments. L3_RS had the highest number of edges (117), followed by L1_RS (108) and L4_RS (100), whereas fewer edges were observed in L2_RS (59) and W_RS (68). With respect to positive and negative associations, L4_RS showed the largest difference between positive and negative edges (54/46).

Proteobacteria and Actinobacteriota were the main hub phyla in all rhizosphere networks. Treatment-related differences were mainly reflected in the presence of additional hub phyla. L4_RS contained the greatest number of hub phyla, including Cyanobacteria, Myxococcota, and Firmicutes, in addition to Proteobacteria and Actinobacteriota. In W_RS, Abditibacteriota and Armatimonadota were identified as hub phyla. Bacteroidota and Firmicutes occurred as hub phyla in L1_RS, whereas Deinococcota and Cyanobacteria appeared in L2_RS and L3_RS, respectively.

Overall, the phyllosphere networks showed clearer changes in the balance between positive and negative associations across shading treatments, particularly under unshaded conditions. In the rhizosphere, network differences were mainly reflected in edge number and the composition of hub taxa at the phylum level among treatments.

## 4. Discussion

### 4.1. Phyllosphere Bacteria Showed Stronger Taxonomic Turnover in Response to Shading Treatments

In this study, the phyllosphere bacterial community showed a stronger response to light variation than the rhizosphere community, reflected primarily in shifts in dominant taxa. This pattern aligns with the ecological characteristics of the leaf surface, which is directly exposed to changes in radiation, temperature, humidity, and ultraviolet intensity. These environmental fluctuations can strongly influence microbial colonization, persistence, and community assembly on leaves [29]. Compared with the rhizosphere, the phyllosphere is also more nutrient-limited and lacks the buffering capacity of the soil matrix, which may further contribute to the pronounced compositional changes observed across the shading gradient.

Among the dominant phyllosphere genera, *Delftia* was highly abundant under several artificial shading treatments, particularly L1_L, L3_L, and L4_L (Figure 3b). A similar dominance pattern has been reported in Brassicaceae crops, where *Delftia* was also identified as a core phyllosphere taxon [30]. Some *Delftia* strains have been reported to promote plant growth, enhance stress tolerance, and suppress fungal pathogens [31,32]. In this study, the consistent dominance of *Delftia* across contrasting light conditions suggests a broad ecological tolerance and strong colonization capacity on leaf surfaces. However, because these findings are derived from amplicon sequencing data, the ecological function of *Delftia* in the phyllosphere of *M. oleifera* requires validation through strain isolation and functional assays. Functional predictions further indicated that phyllosphere microbial profiles varied under full-light conditions (Figure 5 and Figure 6). In L4_L, COG category P, associated with inorganic ion transport and metabolism, showed relatively higher predicted abundance (Figure 5c). This functional category is associated with ion homeostasis, osmotic regulation, and cellular stress adaptation, which may contribute to microbial persistence under fluctuating or stressful environmental conditions [33]. Meanwhile, co-occurrence network analysis revealed that L4_L had a higher proportion of negative associations than shaded treatments (Figure 7d), indicating altered co-variation patterns among phyllosphere taxa under full light. The leaf surface represents a harsh and resource-limited habitat, where microorganisms are directly exposed to radiation, temperature fluctuations, desiccation, and limited nutrient availability. Such conditions may strengthen environmental filtering and promote divergent ecological responses among bacterial taxa, which may partly explain the increased negative associations observed in the L4_L network [34,35]. This network structure was consistent with reduced diversity and increased dominance of *Delftia*, suggesting that full-light exposure was associated with a more uneven and selectively structured community assembly. However, because these networks were inferred using correlation-based methods and a limited number of replicates (*n* = 3), they should be interpreted as preliminary association patterns rather than as evidence of direct microbial interactions.

L2_L exhibited a distinct phyllosphere composition characterized by reduced dominance of *Delftia* and increased relative abundance of actinobacterial genera such as *Actinomycetospora*, *Quadrisphaera*, and *Klenkia* (Figure 3b). Members of Actinobacteria are recognized for their metabolic versatility and their capacity to utilize diverse substrates through the production of extracellular enzymes [36]. Their increased abundance suggests a potential competitive advantage under moderate shading conditions. COG functional predictions indicated enrichment of categories K, L, C, G, and F (Figure 5c), which are associated with transcription, replication, energy production, and nutrient metabolism. These patterns suggest that the L2_L phyllosphere community had a more balanced taxonomic structure and potentially higher functional versatility, possibly reflecting more diversified resource utilization among coexisting taxa under moderate shading.

The field-grown phyllosphere samples differed from all artificial shading treatments. W_L showed relatively high Shannon diversity, although Chao richness was not the highest (Figure 1a,b), indicating a more even rather than more species-rich community. Genera such as *Sphingomonas*, *Amnibacterium*, and *Methylobacterium–Methylorubrum* were more abundant in W_L, whereas *Delftia* was less dominant (Figure 3b). Unlike nursery-grown seedlings, field-grown plants are exposed to more heterogeneous environmental conditions, including surrounding vegetation, microclimatic variability, native soil microbial reservoirs, and diverse airborne inocula, all of which may contribute to the distinct phyllosphere community structure observed in W_L.

Shading primarily altered the relative dominance of key phyllosphere taxa rather than causing uniform increases or decreases in bacterial abundance. Full-light conditions promoted a more uneven community dominated by *Delftia*, whereas moderate shading reduced this dominance and favored a broader range of actinobacterial taxa. These results suggest that appropriate shading management may help maintain a more balanced and potentially functionally diverse phyllosphere bacterial community during *M. oleifera* seedling cultivation.

### 4.2. The Rhizosphere Bacterial Community of M. oleifera Seedlings Mainly Responded to Shading Treatments Through Abundance Adjustment

Compared with the phyllosphere, the rhizosphere bacterial community maintained higher diversity and richness across shading treatments and in field conditions (Figure 1). This pattern is consistent with the buffered nature of the rhizosphere, where the soil matrix can reduce direct fluctuations in temperature and moisture associated with light variation, while root exudates may provide continuous nutrient inputs. In this study, shading did not result in a clear replacement of dominant rhizosphere taxa, such as Proteobacteria. Instead, responses were primarily reflected in changes in the relative abundance of shared dominant groups (Figure 3c,d). Similar patterns have been reported in tomato and other plant systems, where rhizosphere communities are generally more stable than phyllosphere communities and tend to differ mainly in abundance rather than taxonomic turnover [37,38]. The relatively high Shannon and Chao indices in L2_RS further suggest that moderate shading may help sustain rhizosphere bacterial diversity (Figure 1c,d).

The higher relative abundance of *o_Chloroflexales* in L2_RS further reflected compositional differentiation under moderate shading (Figure 4b). Members of Chloroflexales are typically slow-growing bacteria associated with oligotrophic conditions and the degradation of complex organic substrates. Their increased abundance in L2_RS may indicate a relatively stable rhizosphere environment under moderate shading. However, given the limited number of sequencing replicates, this pattern should be interpreted cautiously as a preliminary indication of community variation, and its ecological relevance in *M. oleifera* seedlings requires further validation. At higher taxonomic levels, Proteobacteria and Actinobacteriota remained dominant across all treatments (Figure 3c), consistent with commonly observed patterns in plant-associated microbiomes [39]. However, differences at lower taxonomic levels, especially among genera, better reflected the response of the rhizosphere community to shading. Among the detected genera, *Bacillus* and *Bradyrhizobium* showed treatment-dependent shifts. With decreasing shading intensity, *Bacillus* abundance gradually declined and reached its lowest level under full light, whereas *Bradyrhizobium* showed an opposite trend, increasing under higher light exposure (Figure 3d). In L4_RS, predicted functional profiles based on COG analysis indicated lower relative abundance of category J and higher abundances of categories C and T (Figure 5d), suggesting that functional potential related to translation, energy metabolism, and signal transduction may have shifted under full-light conditions. KEGG-based predictions further showed higher predicted representation of pathways involved in aromatic compound metabolism in L4_RS, including benzoate degradation, degradation of aromatic compounds, and phenylalanine metabolism (Figure 6b,d). Full-light conditions may alter photosynthetic carbon allocation, root development, and the composition of root-derived substrates, thereby affecting the functional potential of rhizosphere bacteria. However, the higher weighted NSTI values observed in the rhizosphere than in the phyllosphere indicate that the rhizosphere predictions were based on more distant reference genomes. Therefore, the KEGG patterns observed in L4_RS are better interpreted as a putative functional shift associated with full-light conditions, rather than as direct evidence of increased aromatic compound degradation activity. To strengthen the functional interpretation of these predictions, future studies integrating metagenomic sequencing, functional gene quantification, or culture-based assays would be valuable, as such approaches have been widely used to link microbial composition with functional potential in soil ecosystems [40].

Compared with artificial shading treatments, the field-grown rhizosphere community exhibited higher relative abundances of *Bacillus* and *norank_f_Xanthobacteraceae*, both of which were also identified in LEfSe analysis (Figure 4b). Certain *Bacillus* species are known for nutrient mobilization, plant growth promotion, and antagonistic activity against soilborne microorganisms [15]. Members of Xanthobacteraceae are also involved in nutrient cycling, and several taxa within this family, such as *Azorhizobium caulinodans*, possess nitrogen-fixing capacity [41]. COG categories associated with amino acid transport and metabolism and transcription showed higher predicted abundance in W_RS than in artificial shading treatments (Figure 5d).

These findings are particularly relevant given the hemiparasitic nature of *M. oleifera*. Previous studies have shown that this species can form haustorial connections with host plant roots and may benefit from nitrogen-fixing hosts [42]. In general, hemiparasitic plants often preferentially associate with nitrogen-fixing or nutrient-rich hosts to meet their nitrogen requirements [43]. Therefore, nitrogen availability likely plays an important role in seedling development. The higher abundance of *Bacillus* and Xanthobacteraceae-related taxa in W_RS, together with enrichment of amino acid metabolism- and transcription-related functional categories, suggests that field conditions may be associated with a more complex nutrient-acquisition environment compared with controlled shading treatments.

### 4.3. Implications for Shading Management and Microbial Resource Exploration in M. oleifera Seedlings

The observed bacterial community differences across shading treatments provide useful insights for optimizing nursery cultivation of *M. oleifera* seedlings from a microecological perspective. Under full-light conditions, the phyllosphere community showed reduced diversity, stronger dominance of *Delftia*, and a higher proportion of negative associations among OTUs. The rhizosphere community exhibited shifts in several plant-associated genera and predicted functional profiles, particularly those related to energy metabolism, signal transduction, and aromatic compound degradation. These patterns suggest that full light was associated with a more selectively structured microbial environment in young seedlings, especially in the phyllosphere, where microorganisms are directly exposed to radiation, surface desiccation, and fluctuations in boundary-layer humidity. Shaded treatments, particularly moderate shading, were associated with higher diversity and a more balanced community structure, indicating that appropriate shading may help maintain a more stable plant-associated bacterial assemblage during seedling development.

These results align with previous reports showing that *M. oleifera* seedlings are sensitive to light availability and may perform better under moderate shading during early growth stages. In this study, shading treatments established a clear PAR gradient, whereas air temperature and soil moisture remained relatively comparable across treatments. Therefore, the observed microbial differences are primarily due to variation in light availability. However, shading may also indirectly affect leaf surface microenvironments, which are particularly important for phyllosphere microorganisms inhabiting nutrient-limited and exposed habitats. This may partly explain the stronger responsiveness of phyllosphere communities compared with rhizosphere communities. Although this study focused on microbial community patterns rather than plant performance, the observed microbial shifts provide a foundation for future research linking shading management, seedling establishment, and plant-associated microbiota. Integrated studies combining microbial community analysis with growth traits, physiological stress indicators, and fine-scale microenvironmental measurements would help elucidate how shading-driven microbial variation contributes to seedling adaptation and performance.

In addition to cultivation practices, the identification of key plant-associated bacterial taxa highlights potential microbial resources for future exploration. Plant growth-promoting bacteria (PGPB) have attracted increasing attention due to their roles in nutrient acquisition, stress tolerance, pathogen suppression, and sustainable crop production, although their effects are often context-dependent and influenced by host identity and environmental conditions [44]. In this study, *Bacillus* was relatively abundant in the rhizosphere. Many *Bacillus* species are well-characterized PGPBs with functions including nutrient mobilization, phytohormone production, enhancement of stress tolerance, and suppression of soilborne pathogens [45,46,47]. These characteristics suggest that *Bacillus* may represent a promising candidate group for future isolation and functional evaluation in *M. oleifera* seedling systems.

Future microbial resource development for *M. oleifera* should prioritize taxa with strong rhizosphere colonization capacity, nutrient acquisition potential, and adaptability to nursery environments. In addition to *Bacillus*, arbuscular mycorrhizal fungi may be considered due to their well-established roles in improving root establishment and enhancing water and mineral uptake. Functional fungi such as *Trichoderma* may also represent valuable candidates, as this genus has been reported in the endophytic fungal community of *M. oleifera* and is widely recognized for promoting root growth and suppressing plant pathogens [48,49,50,51]. These results suggest that moderate shading combined with the application of locally adapted beneficial microorganisms may represent complementary strategies for improving *M. oleifera* seedling cultivation and establishment. Future studies should further validate these hypotheses through targeted strain isolation, inoculation experiments, and multi-omics or functional gene-based approaches under controlled shading conditions. Moreover, although the 97% OTU-based clustering approach was suitable for identifying broad treatment-related patterns in this study, future work using ASVs, such as those generated by DADA2, would enable higher-resolution assessment of fine-scale taxonomic responses of *M. oleifera*-associated bacterial communities to shading gradients.

## 5. Conclusions

This study demonstrated that bacterial communities associated with *M. oleifera* seedlings respond differently in the phyllosphere and rhizosphere. The phyllosphere bacterial community showed lower α-diversity and higher sensitivity to shading treatments, with compositional shifts mainly driven by changes in dominant taxa, including Proteobacteria- and Actinobacteriota-related groups. The rhizosphere maintained higher diversity and a larger shared bacterial pool, with treatment effects primarily reflected in changes in relative abundance. Predicted functional profiles and co-occurrence network analyses further suggested that the phyllosphere was more responsive to the shading gradient, whereas the rhizosphere exhibited higher compositional and less pronounced variation in predicted functional profiles. Full-light conditions were associated with reduced phyllosphere diversity, increased dominance of specific taxa, and altered bacterial association patterns. Moderate shading supported a more balanced and less uneven bacterial assemblage in the phyllosphere. These results highlight the contrasting responses of the phyllosphere and rhizosphere to shading and provide a microecological basis for shading management during *M. oleifera* seedling cultivation.

## Data Availability

The raw data supporting the findings of this study are not publicly available at this stage because they are part of ongoing analyses and related unpublished work.

## Supplementary Materials

The following supporting information can be downloaded at: https://www.mdpi.com/article/10.3390/microorganisms14071421/s1, Table S1: Pairwise comparisons of Shannon and Chao indices among phyllosphere bacterial communities of *Malania oleifera* seedlings under different light treatments; Table S2: Pairwise comparisons of Shannon and Chao indices among rhizosphere bacterial communities of *Malania oleifera* seedlings under different light treatments; Table S3: Weighted NSTI values for PICRUSt2 functional prediction of phyllosphere and rhizosphere bacterial communities.
